# Supplementary material for: Agile THz-range spectral multiplication of frequency combs using a multi-wavelength laser
Source: Nat Commun. 2024 Feb 12;15:1305. doi: 10.1038/s41467-024-45610-7 (PMC10861570; doi:10.1038/s41467-024-45610-7)
Supplement: Supplementary file 1 — Supplementary Information [file 41467_2024_45610_MOESM1_ESM.pdf]

Agile THz-range spectral multiplication of frequency combs using a multi-wavelength laser

- Supplementary Information -

SHAHAB ABDOLLAHI[†], MATHIEU LADOUCE, PABLO MARIN-PALOMO, MARTIN VIRTE[‡]

[†]*mohammadshahab.abdollahi@vub.be*, [‡]*martin.virte@vub.be*
Brussels Photonics Team (B-PHOT), Vrije Universiteit Brussel, Pleinlaan 2, 1050, Brussel, Belgium

1. Mode switching in feedback-controlled MWL without optical injection

Figure S1(a) depicts the sketch of the photonic integrated circuit (PIC) employed in our experiments consisting of the multi-wavelength laser (MWL) and the feedback cavity. The MWL cavity is defined, on one end, by the broadband reflector (BR) and, on the other end, by each of the distributed Bragg reflectors (DBR_1 and DBR_2), effectively outlining two cavities. In this section, we characterize the emission of the MWL under feedback control but without optical injection. The feedback cavity, monolithically integrated with the MWL, allows us to control the laser output and achieve switching between emissions at different longitudinal modes. The emission control is achieved by adjusting the corresponding feedback strength and feedback phase, by controlling the current I_{SOA2} applied to the semiconductor optical amplifier (SOA_2) and the voltage V_{EOPM} supplied to the electro-optic phase modulator (EOPM, $V_\pi \approx 12$ V), respectively. For $I_{\text{SOA2}} = 0$ mA, the feedback strength is weak enough to assume no signal is fed back to the MWL.

We consider the same configuration as used in the main paper with $I_{\text{SOA1}} = 30$ mA, $I_{\text{DBR1}} = 0$ mA, $I_{\text{DBR2}} = 1$ mA. Without feedback, this configuration leads to emission at $\lambda_4 = 1547.5$ nm, while emission at the other wavelengths, i.e., $\lambda_1 = 1536.67$ nm, $\lambda_2 = 1536.9$ nm, and $\lambda_3 = 1537.12$ nm is suppressed, as shown in Fig. S1(b). The longitudinal modes at λ_1, λ_2, and λ_3 are linked to the cavity defined by DBR_1, while λ_4 is associated to the cavity defined by DBR_2. When "turning on" the feedback, setting $I_{\text{SOA2}} = 21$ mA, switching between emission at λ_1, λ_2 and λ_4 can be achieved by adjusting V_{EOPM}, see Fig. S1(c). For V_{EOPM}<1.7 V, the laser emits at λ_1 while

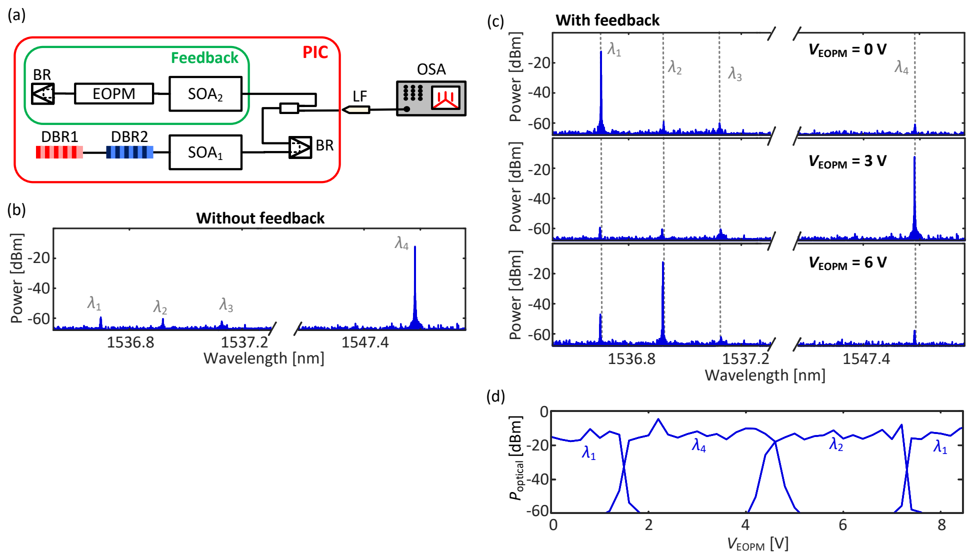

Fig. S1. Mode switching in our feedback-controlled MWL. (a) Photonic integrated circuit (PIC) including the MWL and the monolithically integrated feedback cavity. The lensed fiber (LF) collects the light out of the chip for spectral measurements with an optical spectrum analyzer (OSA). (b) Optical spectrum of the MWL emission without feedback, for $I_{\text{SOA2}} = 0$ mA. (c) Optical spectra of the MWL emission with feedback, for $I_{\text{SOA2}} = 21$ mA, and different V_{EOPM} values. (d) Optical power of each of the MWL modes as a function of V_{EOPM}. The switching happens between three modes of the MWL: λ_1, λ_4, and λ_2. In all EOPM voltage values, λ_3 is strongly suppressed and it is not affected by feedback.

the other longitudinal modes are suppressed. By further increasing the voltage, the emission is switched to λ_4. For 4.2 $V < V_{EOPM} < 5.2$ V, emission at λ_4 is "turned off" and the laser switches to emit at λ_2. Figure S1(d) shows the power emitted by the MWL at around the wavelengths λ_1, λ_2, λ_4 for V_{EOPM} values between 0 V and 8.5 V. For all V_{EOPM} values, the MWL mode emitting at λ_3 is suppressed, i.e., the power around λ_3 remains more than 30 dB below the power of the dominant mode, and it is not influenced by feedback. For this reason, we chose to inject the optical frequency comb around λ_3.

2. Spectral evolution of re-generated and multiplied comb for different values of κ_{inj}, Δ, and number of lines of the injected comb

In this section, we provide additional information on the impact of the injection strength κ_{inj}, detuning Δ, and the number of comb lines on the spectral multiplication process. Detailed investigations however remain to be performed to fully clarify the reported behavior and underlying mechanism.

Here, the laser configuration is slightly different from that used in the main paper with $I_{SOA1} =$ 35 mA, $I_{DBR1} = 0$ mA, and $I_{DBR2} = 0$ mA. Without the injection, the MWL emits again at four longitudinal modes, which we label I, II, III, and IV to avoid confusion with the main case reported. Optical power of each mode is -13.6 dBm (dominant mode), -43.4 dBm, and -34.7 dBm, and -47.2 dBm respectively. No optical feedback is considered here.

First, We inject a frequency comb with five tones and an FSR of 1 GHz. We sweep the center frequency of the injected frequency comb around the longitudinal mode (II), and consider two different injection strength values, $k_{inj} = 4.5$ dBm and $k_{inj} = 9.5$ dBm. Figure S2(a), shows the spectral evolution of the re-generated and multiplied combs when sweeping the comb around (II). We observe not only comb multiplication around the dominant mode (I), but also comb multiplication around two strongly suppressed modes: III and IV. By increasing the injection strength from 4.5 dBm to 9.5 dBm, the bandwidth and power evolution of the re-generated and multiplied combs appear to be different when the detuning is varied, see the second column of Fig. S2(a). Second, we perform the same experiment, but with a frequency comb with 15 tones and a 1 GHz FSR injected around mode (II); result is shown in Figure S2(b). By increasing the numbers of comb lines and for low injection strength, the multiplied comb only appears around the dominant mode (I), and the other suppressed mode are not triggered, see the first column of Fig. S2(b). On the other hand, by increasing the injection strength and sweeping the comb around (II), the other suppressed modes, i.e., (III) and (IV) start emitting with the same modulation as the injected comb, see the second column of Fig. S2(b).

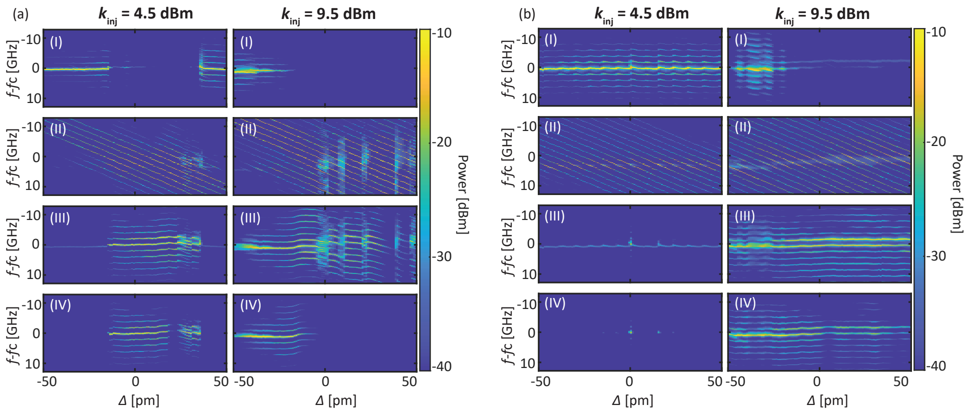

Fig. S2. Spectral evolution of the re-generated and multiplied combs as a function of detuning Δ for different injection strengths κ_{inj} and different comb line numbers of the injected comb. (a) Frequency comb with 5 tones and 1 GHz FSR is swept around mode (II) with two different injection strengths: $\kappa_{inj} = 4.5$ dBm on the left and $\kappa_{inj} = 9.5$ dBm on the right. The injection leads to a comb regeneration around mode (II) and comb multiplications around the dominant mode (I) and two strongly suppressed modes, (III) and (IV). (b) Frequency comb with 15 tones and 1 GHz FSR is swept around mode (II). At low injection strength, the multiplied comb only appears around mode (I), while by increasing the injection strengths, modes (III) and (IV) are triggered and show the same modulation as the injected comb.

3. Evolution of the bandwidth and optical power of the re-generated and multiplied comb with the FSR of the injected comb

In this section, we look at the effect of the FSR of the injected comb on the quality of the re-generated and multiplied combs. The laser configuration is identical to the one used in the main paper with $I_{\mathrm{SOA1}} = 30$ mA, $I_{\mathrm{DBR1}} = 0$ mA, and $I_{\mathrm{DBR2}} = 1$ mA. With this injection current and without feedback, the laser emission is dominated by the mode at λ_4 and the other longitudinal modes are strongly suppressed, as detailed in Section 1. We inject a frequency comb with five tones around λ_3 with different FSRs of 500 MHz, 1 GHz, or 3 GHz, and record the evolution of the re-generated and multiplied combs when scanning the detuning value, see Fig. S3 for the measured spectral evolution. By sweeping the center wavelength $\lambda_c = c/f_c$ of the frequency comb around λ_3, a re-generated comb with larger bandwidth and higher optical power is emitted at detuning values close to the wavelength λ_3 of the injected mode. In addition, a multiplied comb is emitted around the wavelength λ_4 of the un-injected mode, 1.3 THz away from the injected mode. At detuning values close to the wavelength λ_3 of the injected mode, i.e., for $\Delta \approx 0$, the re-generated comb experiences different dynamical behavior while the multiplied comb is suppressed and exhibit a reduced bandwidth. When increasing the FSR of the injected comb, a periodicity with the detuning appears, see Fig. S3(c). The overall comb bandwidth seems approximately fixed, thus leading to a smaller number of comb lines as the FSR is increased. We associate this behaviour with the limited relaxation oscillation frequency of the laser.

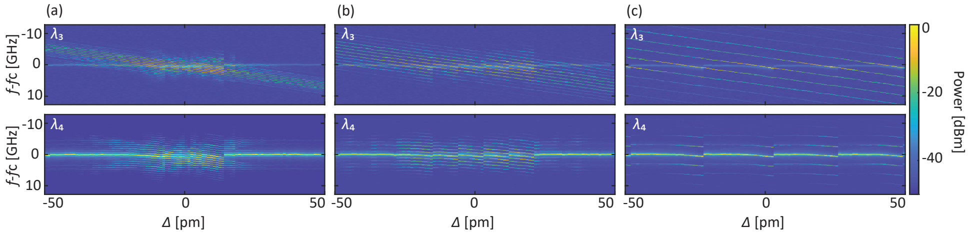

Fig. S3. Spectral evolution of the re-generated comb (emitted around λ_3) and multiplied comb (emitted around λ_4) as a function of the detuning, i.e., the difference between the wavelength c/f_c of the central tone of the injected comb and λ_3, for FSR values of (a) 500 MHz, (b) 1 GHz, and (c) 3 GHz. The injected optical frequency comb consists of 5 tones with approximately similar optical power.

4. Feedback-controlled high-speed mode switching in an MWL

In this section, we briefly discuss the experimental prodecure that we followed to measure the switching time, i.e., the time it takes the laser to switch mode emission when varying the feedback phase (via V_{EOPM}) of the feedback signal. The switching time τ_s includes the reaction time τ_r, i.e., the time the MWL needs to react to the change of feedback signal due to a change of the applied V_{EOPM}. We consider the same MWL and feedback configuration as in the main manuscript and we monitor the switching between the modes emitting at $\lambda_1 = 1536.70$ nm and $\lambda_4 = 1547.58$ nm, occurring at around $V_{\text{EOPM}} = 0.6$ V. Figure S4(a) describes the setup used to measure the switching time. The setup consists of an arbitrary waveform generator (Keysight M9502A, 50 GHz bandwidth, 120 GSa/s) that generates a step function which is added through a bias tee to a DC signal, generated by a voltage source (Agilent E3646A). We apply a step function with a pulse width of 100 ns, a rise and fall time of 100 ps, and a peak-to-peak amplitude of 0.5 V. A DC voltage of 1.5 V is applied to the EOPM of the MWL via the bias tee. The emitted light is coupled into the lensed fibre after which an optical bandpass filter (EXFO XTM-50) selects one of the two wavelengths for optical power measurements using an oscilloscope (Tektronix CSA 7404, 4 GHz, 20 GSa/s) with a built-in photodiode.

The delay between the electronic trigger generated by the AWG and the measurement is conservatively estimated at 221 ns based on the electrical and optical path length of the signal and compensated in the experimental results. The current setup only allows us to monitor one wavelength at a time. We show here the turn off dynamics of λ_1 as the laser switches from λ_1 emission to λ_4. The experimental results are shown in Fig. S4(b) for the falling trigger edge. From left to right, the vertical black dotted lines indicate the time (compensated for the propagation delay) when the V_{EOPM} value croses te switching voltage at around 0.6 V, the time at which the laser starts reacting, $\tau_r = 1.6$ ns, and the time at which the switching is done, $\tau_s = 4.0$ ns, i.e.,when the optical power emitted at λ_1 is reduced by more than 10 dB.

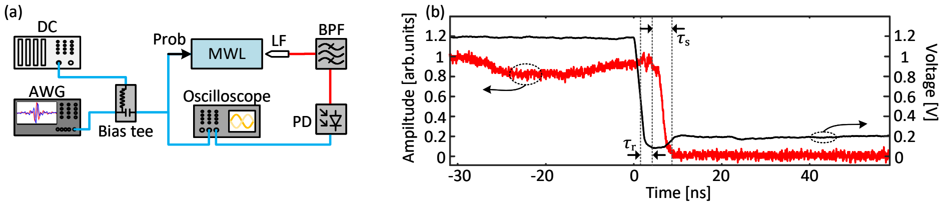

Fig. S4. Investigation of high-speed feedback-controlled mode switching in an MWL (a) Experimental setup for the switching speed measurement: AWG: Arbitrary waveform generator. DC: voltage source. MWL: Multi-wavelength laser. PD: Photodiode. LF: Lensed fiber. BPF: Tunable bandpass filter. Blue lines: coaxial connections. Red lines: optical fiber connections. (b) In black: Voltage signal sent to the EOPM, see the y-axis on the right side of the plot. In red: Signal recorded by the oscilloscope, associated with the power emitted at λ_1, see the y-axis on the left side of the plot. The vertical dotted lines indicates the reaction time, τ_r, and the switching time, τ_s, of the laser

5. Cascaded phase locking dependence on the frequency offset f_{tone} of the side tone

As discussed in the main manuscript, adding an extra tone is a promising technique to achieve cascaded phase locking. We demonstrated that by fine-tuning the frequency offset between the central frequency of the comb and the extra tone we are able to obtain phase correlation between the three neighboring combs emerged in the same cavity. In this section, we present the quality of the cascaded phase-locked comb outside the optimized range of f_{tone} values and highlight the fact that the accurate optimization of the f_{tone} is crucial to achieving cascaded phase locking. Figure S5(a), shows the optical spectrum of the MWL laser under comb injection (5 tones, 1 GHz FSR) around λ_3, while the extra tone is injected close to λ_2 at $f_{tone} = 26.1$ GHz. The injection leads to the comb re-generation at the injected mode, λ_3, and comb multiplication at λ_2. The interaction between the re-generated and multiplied combs leads to a modulation signal at the beating frequencies of the two combs, prompting the emission of a frequency comb around λ_1, which can be phase-locked to the other two frequency combs. At $f_{tone} = 26.1$ GHz, the resulting beating is not well aligned with the modulation caused by the injection itself leading to broad optical linewidths, see the inset in Fig. S5(a). Figure S5(b) shows the RF spectrum of the resulting beating between the multiplied comb centered at λ_2 and the low-power comb emitted at λ_1. By zooming in at one of the RF peaks, we observe a broad peak appearing around the beat note, this is an indication of the poor coherence between the two multiplied combs.

As discussed in the main manuscript, the optical linewidth can be drastically improved and the broadband peak appearing around the beat note can be suppressed by tuning f_{tone}.

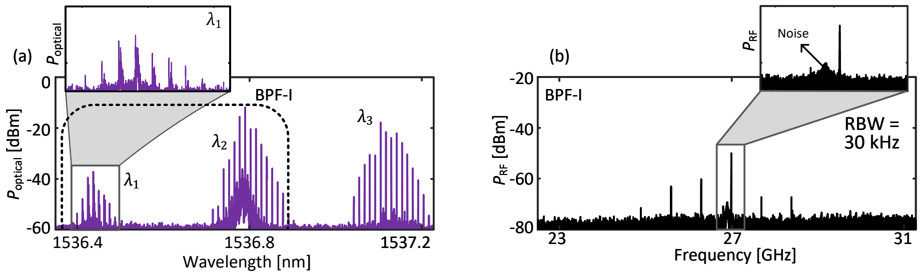

Fig. S5. Phase coherence control of the multiplied combs induced by injecting an additional sideband tone. The frequency offset between the central wavelength of the injected frequency comb and the extra tone is set to $f_{tone} = 26.1$ GHz, corresponding to a "non-optimal" value in which the multiplied comb at around λ_1 is not phase correlated to the other frequency combs. (a) Optical spectrum of the MWL emission featuring the re-generated comb at λ_3 and multiplied combs emitting at around λ_2 and λ_1. The inset depicts a multiplied comb featuring comb lines with low optical power and large linewidth, in contrast with the high power and low linewidth obtained for optimized f_{tone} values described in Fig. 4 of the main paper. (b) RF spectrum resulting from the beating of the combs emerging at around λ_1 and λ_2. The inset shows the co-existence of a broadband noise component, indicating the poor phase coherence between the corresponding frequency combs.

6. Dynamical behavior of the MWL under frequency comb injection

In panel (3) of the Fig. 5 of the main paper, the black dots signal different dynamical behavior experienced by both injected and un-injected modes.
To clarify the dynamical states and explain how they can be differentiated from the normal states, we discuss here two additional simulations using the same parameters as in the main manuscript. In Fig. S6(a), we give an example of the time evolution and its corresponding spectrum of the re-generated and multiplied signal when considering $\kappa_{inj} = 0.07$ and $\Delta = 9.56$ GHz, falling in the white region of panel (3) of the Fig. 5 of the main paper. The periodic time series and the clear emergence of a frequency comb around both modes with the FSR of 1 GHz highlights the fact that the laser operates at the steady state and no dynamics are observed at this region. By changing the injection strength and detuning, different forms of dynamics can appear around the injected and un-injected modes. By choosing $\kappa_{inj} = 0.06$ and $\Delta = -13.055$ GHz, we make the laser operate within the black region of panel (3) of the Fig. 5 of the main paper. The time series and the optical spectrum computed at this operating point show different dynamical behavior experienced by both injected and un-injected modes, see Fig. S6(b).

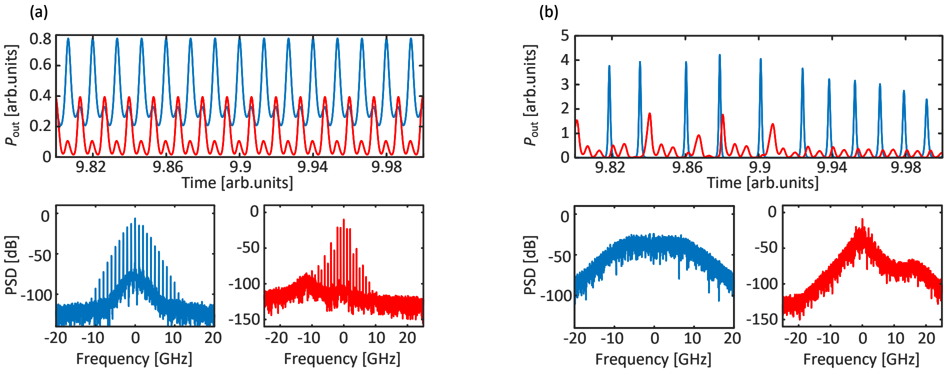

Fig. S6. Numerical simulation of the time evolution and the optical spectrum of the output signal emerging around the injected and un-injected modes. (a) The time series (first raw) and the optical spectra (second raw) of the injected (blue) and un-injected (red) output signals when a comb with 5 tones and the FSR of 1 GHz is injected around the suppressed mode with $\kappa_{inj} = 0.07$ and $\Delta = 9.56$ GHz. The time series shows a periodic oscillation with a 1 GHz repetition rate, and the corresponding optical spectra preserve the FSR of the original comb highlighting the fact that the quality of the injected comb is preserved and transferred to the un-injected mode (b) Time series and optical spectra of the injected (blue) and un-injected (red) output signals at $\kappa_{inj} = 0.06$ and $\Delta = -13.055$ GHz. Such injection parameters leads to different dynamical behavior of the MWL experienced by both injected and un-injected modes.

7. Numerical simulations showing spectral comb multiplication with feedback-control

As a complement to our main manuscript, we show here that the multiplied comb switching triggered by optical feedback is well reproduced by the rate equation model.
Here, we extend the model to include three modes in which two modes are dominant with modal gains $g_1 = 0.995$ and $g_2 = 0.9945$. The third mode is strongly suppressed with $g_3 = 0.88$. The equations read as:

$$\frac{dE_1}{dt} = (1+i\alpha)(g_1 N_1 - \frac{1-g_1}{2})E_1 + \kappa e^{-i\theta_1} E_1(t-\tau) \tag{S1}$$

$$\frac{dE_2}{dt} = (1+i\alpha)(g_2 N_2 - \frac{1-g_2}{2})E_2 + \kappa e^{-i\theta_2} E_2(t-\tau), \tag{S2}$$

$$\frac{dF_3}{dt} = (1+i\alpha)(g_3 N_3 - \frac{1-g_3}{2})F_3 + \kappa e^{-i\theta_3} F_3(t-\tau)e^{-i\Delta\tau} + \kappa_{\text{inj}} m(t) - i\Delta F_3, \tag{S3}$$

$$\frac{dN_1}{dt} = P - N_1 - (1+2N_1)(g_1|E_1|^2 + g_2\beta|E_2|^2 + g_3\beta|E_3|^2), \tag{S4}$$

$$\frac{dN_2}{dt} = P - N_2 - (1+2N_2)(\beta g_1|E_1|^2 + g_2|E_2|^2 + g_3\beta|E_3|^2), \tag{S5}$$

$$\frac{dN_3}{dt} = P - N_3 - (1+2N_3)(\beta g_1|E_1|^2 + \beta g_2|E_2|^2 + g_3|E_3|^2). \tag{S6}$$

We set the pump parameter $P = (J - J_{\text{th}})/2J_{\text{th}} = 0.5$, corresponding to an injection current 2 times the laser threshold. The cross-saturation parameters are all fixed to the same value $\beta = 0.99$. The carrier lifetime is $\tau_n = 1000$, normalized by the photon lifetime. The linewidth enhancement factor, which is represented as α, is set to $\alpha = 3$.
The feedback control is modeled by the second term of the field equations, Eqs. S1-S3, already explained in the Methods of the main paper. The feedback rate is set to $\kappa = 0.006$ and the time delay to $\tau = 100$, normalized by the photon lifetime. A variable feedback phase Φ term mimicking the effect of the EOPM is applied to all three modes. The feedback phases $\theta_{1,2,3}$ for each wavelength are therefore set to $\theta_1 = \Phi$, $\theta_2 = \Phi + \pi$ and $\theta_3 = \Phi$, with a π phase shift between the two dominant modes for the feedback control. In practice, similar behaviors are observed in a relatively wide range of parameters.
We inject the narrow comb, $m(t)$, with 5 tones and a normalized FSR of 0.019. around the suppressed mode of our three-wavelength laser model, i.e. around mode 3. As such, Eq. S3 is written for $F_3(t) = E_3(t)e^{-i\Delta t}$, i.e., the electrical field E_3 shifted in frequency by the detuning Δ to make the equation autonomous. We set the detuning to $\Delta = 0.05$ and the injection strength to $\kappa_{\text{inj}} = 0.05$. Figure S7(a) shows the spectral evolution of the re-generated and multiplied combs while Φ varies from 0 to 2π. The re-generated comb is not affected by feedback showing a continuous emission, while the multiplied comb switches from mode 1 to mode 2. In Fig. S7(b), we give the optical spectra of the re-generated and multiplied combs at $\Phi = \pi/2$ (first row) and $\Phi = 3\pi/2$ (second row). Apart from the emergence of dynamics, our numerical analysis shows the same switching behavior as in the experiment. To link with the experimental observations, in Fig. S7 we have expressed the Fourier frequency in Hertz by assuming a photon lifetime of 3 ps. For this photon lifetime, the FSR of the injected comb corresponds to 1 GHz, while the detuning corresponds to 2.65 GHz.

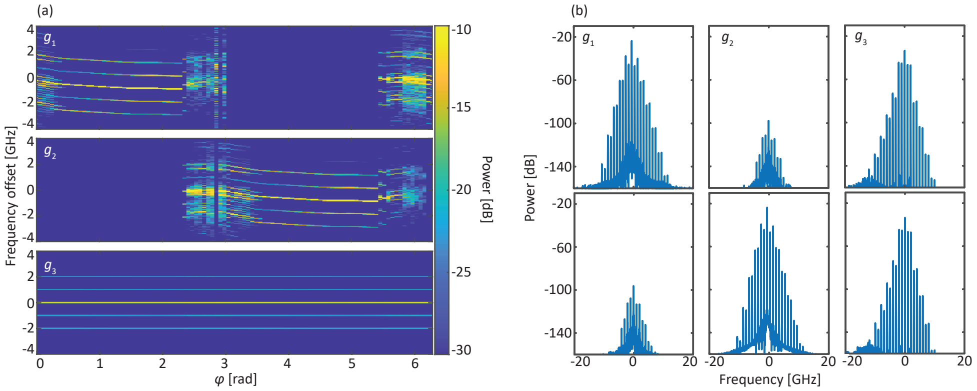

Fig. S7. Programmable spectral multiplication of a three-mode laser subject to frequency comb injection and feedback. (a) spectral evolution of the re-generated (g_3) and multiplied combs ($g_{1,2}$), highlighting the switching capability between the two multiplied combs by applying feedback. Before the injection, by choosing $g_1 = 0.995$, $g_2 = 0.9945$, and $g_3 = 0.88$, the laser is dominated by $g_{1,2}$ and the third mode is suppressed by more than 50 dB. A frequency comb with 5 tones and 1 GHz FSR is injected around g_3. The feedback strength, time delay, and feedback phase are chosen to enable the switching between the two multiplied combs while the injected comb is not affected by feedback: $\kappa = 0.006$, $\tau = 100$, $\theta_1 = \Phi$, $\theta_2 = \Phi + \pi$, and $\theta_1 = 0$, where Φ varies from 0 to 2π. (b) Optical spectrum of the re-generated and multiplied combs at $\Phi = \pi/2$ and $\Phi = 3\pi/2$ corresponding to the first and second rows, respectively.

8. 176 GHz and 45 GHz-range frequency comb spectral multiplication

In this section, we demonstrate frequency comb spectral multiplication at 45 GHz and 176 GHz frequency offsets by utilizing an MWL in which the two DBR structures feature closely spaced spectral passbands. The Bragg wavelengths of the DBRs are separated by approximately 1 nm. In this structure, the BR from Fig. S1 in Section 1, is replaced by a third DBR (DBR3) to achieve better mode selectivity. Setting $I_{\mathrm{SOA1}} = 60$ mA, $I_{\mathrm{DBR1}} = 15.2$ mA, $I_{DBR2} = 4.9$ mA, and $I_{\mathrm{DBR3}} = 8.6$ mA, and "turning on" the feedback by setting $I_{\mathrm{SOA2}} = 15.2$ mA and $V_{\mathrm{EOPM}} = 0$ V, the laser emission is dominated by the longitudinal mode at λ_1, associated with DBR1, while the longitudinal modes at λ_2 and λ_3 , linked to DBR2, are strongly suppressed. We inject an optical frequency comb with 5 comb lines and an FSR of 1 GHz around λ_3, which is strongly suppressed before the injection. We set the detuning and injection strength at $\Delta = 3.62$ GHz and $\kappa_{inj} = 9.5$ dBm, respectively. The comb injection forces the laser to re-generate the frequency comb around the injected mode and multiply the comb around the un-injected modes. Fig. S8(a), represents the spectral evolution of the re-generated and multiplied combs while V_{EOPM} is changed from 0 V to 10 V. At voltage values below 6.8 V, the multiplied comb appears around the longitudinal mode at λ_1. If we keep increasing the voltage applied to the EOPM, the multiplied comb around λ_1 is switched off and the comb around the longitudinal mode at λ_2 is switched on. In the voltage range from $V_{EOPM} = 6.8$ V to $V_{EOPM} = 7.6$ V, the multiplied comb is emitted around the mode at λ_2. If we increase the voltage above 7.8 V, a switching happens between comb emissions around λ_2 and λ_1. Fig. S8(b) shows the optical spectrum of the re-generated and multiplied combs at three different V_{EOPM} values, demonstrating controlled spectral multiplication of a frequency comb with frequency offsets of 45 GHz and 176 GHz approximately. Additionally, we demonstrate simultaneous frequency comb emission around λ_1, λ_2, and λ_3 at a $V_{\mathrm{EOPM}} = 7.8$ V.

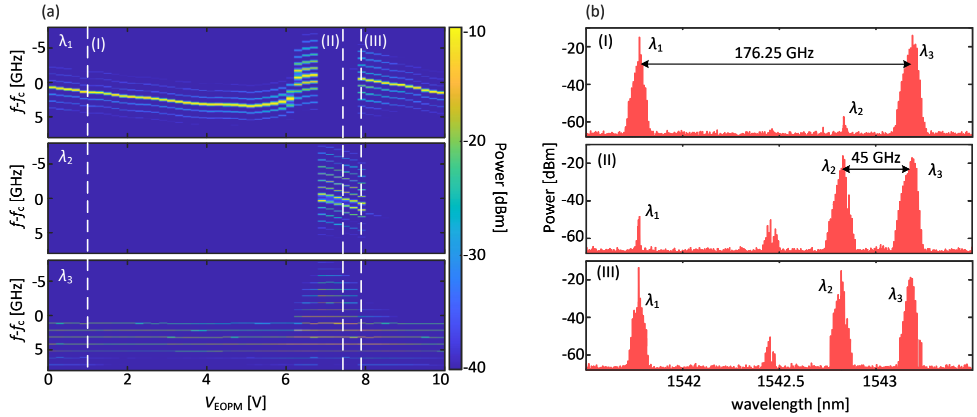

Fig. S8. Controllable comb multiplication using an MWL with 1 nm cavity mode separation (a) spectral evolution of the re-generated and multiplied comb while sweeping the EOPM voltage. The first and second rows indicate the spectral evolution of the multiplied combs appear around λ_1 and λ_2, respectively. The third row shows the spectral evolution of the re-generated comb which appears around the injected mode λ_3. (b) Optical spectrum featuring the re-generated and multiplied combs at 0.8 V, 7.4 V, and 7.8 V (from top to bottom). The corresponding voltages are indicated in panel a by the white dashed lines.
